# Biomass Distribution and Aboveground Carbon Stock in Green Retaining Walls Covered with *Hedera colchica*

**DOI:** 10.3390/plants15193030

**Published:** 2026-10-03

**Authors:** Turan Yüksek, Filiz Yüksek, Gülcay Ercan Oğuztürk, Ebrike Karaca Yüksek, Türker Oğuztürk

**Affiliations:** 1 Department of Landscape Architecture, Faculty of Engineering and Architecture, Recep Tayyip Erdoğan University, Rize 53020, Türkiye; turan.yuksek@erdogan.edu.tr (T.Y.); turker.oguzturk@erdogan.edu.tr (T.O.); 2 Rize Forest Enterprise Directorate, Trabzon Regional Directorate of Forestry, General Directorate of Forestry, Rize 53100, Türkiye; filiz6108@gmail.com; 3 Graduate School of Natural and Applied Sciences, Gazi University, Ankara 06500, Türkiye; karacaebrike@gmail.com

**Keywords:** allometric model, biomass, CO_2_ equivalent, carbon stock, urban green infrastructure, *Hedera colchica*, green retaining wall

## Abstract

Green retaining walls are among the nature-based solutions that can contribute to climate change mitigation by increasing carbon storage capacity in urban areas. This study aimed to determine the aboveground biomass, aboveground carbon stock, and CO_2_-equivalent values of *Hedera colchica* growing on green retaining walls and to evaluate the relationships among these parameters. Branch and leaf components were assessed separately in 16 samples. Oven-dry biomass, dry matter content, aboveground carbon stock, and CO_2_ equivalent were calculated, and relationships among the variables were examined using Pearson correlation and regression analyses. The results showed that the branch component had significantly higher oven-dry biomass (384.08 vs. 219.62 g m^−2^), dry matter content (52.75% vs. 38.35%), aboveground carbon stock (192.04 vs. 109.81 g C m^−2^), and CO_2_-equivalent values (704.79 vs. 403.00 g CO_2_e m^−2^) than the leaf component (*p* < 0.01). A very strong positive relationship was identified between total dry biomass and oven-dry branch biomass (*r* = 0.928, *p* < 0.001), while mean branch diameter showed the strongest relationship with total dry biomass and total aboveground carbon stock (*r* = 0.803, *p* < 0.001). In the regression analyses, linear models based on mean branch diameter explained 59.6% of the variation in branch biomass, branch aboveground carbon stock, and branch CO_2_ equivalent and 64.6% of the variation in the corresponding total values. By contrast, leaf biomass, leaf aboveground carbon stock, and leaf CO_2_ equivalent were better described by power models (*R*^2^ = 0.319). The leaf-to-branch ratio and leaf mass fraction showed significant positive relationships with leaf carbon stock and CO_2_ equivalent, whereas branch mass fraction showed significant negative relationships with these variables. No significant relationships were found between residual moisture content after air drying and carbon-storage indicators. The findings suggest that *Hedera colchica*-covered retaining walls may contribute to aboveground carbon-storage ecosystem services, while mean branch diameter may serve as a useful site-specific morphological indicator for estimating biomass and carbon stock, highlighting the potential contribution of *Hedera colchica*-covered retaining walls to aboveground carbon storage under the site-specific conditions examined in this study.

## 1. Introduction

Rapid population growth and unplanned urbanization have led to the replacement of natural surfaces with impervious construction materials such as concrete, asphalt, and stone, resulting in increased carbon emissions, deterioration of microclimatic conditions, and intensification of the urban heat island effect [1,2,3,4,5,6,7]. Particularly in densely built-up sloping areas, both high building density and the widespread use of retaining walls may increase surface temperatures in the surrounding environment. This situation can produce various adverse effects on highly urbanized ecosystems, especially campus environments [8,9,10,11]. Therefore, within sustainable urban planning approaches developed to address climate change and the urban heat island effect, practices aimed at integrating structural surfaces with vegetated systems have gained increasing importance [12,13].

Vegetation-covered systems contribute to reducing ambient temperatures through mechanisms such as evapotranspiration, shading, moisture retention, and changes in albedo [14,15,16,17,18,19]. At the same time, they provide a carbon-regulation function by contributing to the capture and storage of atmospheric carbon [20,21,22,23]. In particular, twining and climbing plants used on vertical surfaces such as retaining walls can provide nature-based solutions by increasing the amount of vegetated surface area in spatially constrained urban environments [24]. In this context, green walls and vegetated retaining-wall systems not only improve visual quality and provide aesthetic benefits [25], but also deliver multiple ecosystem services, including energy savings [14,26,27], support for biodiversity [28,29], particulate matter capture [30,31], noise reduction [32,33,34], improved air quality, and regulation of microclimatic conditions [35,36,37,38,39]. Quantifying the carbon-storage potential of vertical greening systems is therefore critical for evaluating their climate-regulation functions.

Among climbing plants, species of the genus *Hedera* are widely preferred in landscape planning and implementation, particularly in areas with extensive wall surfaces, because of their evergreen habit, rapid growth, shade tolerance, and ability to attach to different surfaces [40]. Owing to their dense foliage, these species are capable of reducing surface temperatures and have also been reported to possess considerable potential for biomass production and carbon storage [40]. *Hedera colchica*, which is naturally distributed in northeastern Türkiye, is considered suitable for use in vertical greening systems because of its adaptation to high-humidity conditions and its ability to rapidly cover wall surfaces. In woody plants, biomass allocation among plant organs is relevant to carbon-storage assessments because long-lived woody tissues can retain substantial amounts of carbon [41,42,43]. Therefore, organ-level assessment can provide a more detailed understanding of aboveground carbon distribution than estimates based solely on total biomass. Nevertheless, studies on the carbon stock and carbon-storage potential of vegetated retaining walls remain limited. In particular, research quantitatively assessing organ-level biomass distribution, carbon stock, and CO_2_-equivalent values in vertical greening systems established with *Hedera colchica* is scarce. This lack of information limits the quantitative evaluation of the ecosystem services provided by vegetated retaining walls in terms of carbon storage and constrains comparisons with other vertical greening systems.

The high rainfall and humidity conditions of the Eastern Black Sea Region provide a suitable environment for the growth of *Hedera colchica*. Its evergreen growth habit, strong attachment to vertical surfaces, and ability to establish extensive vegetation cover increase its potential for use on vegetated retaining walls. In Rize, where the present study was conducted, numerous retaining walls have been constructed because of the region’s topographic conditions, and a substantial proportion of these structures remain devoid of vegetation. Such unvegetated surfaces not only create visually unfavorable conditions but also lack the potential ecosystem services associated with vegetation cover, such as carbon storage, microclimate regulation, and support for biodiversity. However, quantitative data on the biomass production and carbon-storage potential of retaining walls covered with *Hedera colchica* remain very limited.

The aim of this study was to determine the total aboveground biomass, aboveground carbon stock, and CO_2_-equivalent values per unit area in vegetated retaining walls covered with *Hedera colchica* and to quantify the distribution of biomass and carbon stock between branch and leaf components. In addition, the relationships between biomass characteristics and carbon-stock indicators were evaluated. The novelty of this study lies in providing quantitative, organ-level estimates of aboveground biomass, aboveground carbon stock, and CO_2_ equivalent for *H. colchica* growing on vegetated retaining walls, for which empirical information remains limited. By addressing this knowledge gap, the study contributes to a more detailed understanding of organ-level carbon allocation and the carbon-storage ecosystem services provided by vegetated retaining walls under the humid climatic conditions examined in this study.

## 2. Results

Significant differences were identified between the branch and leaf components of *Hedera colchica* in terms of biomass and carbon-stock parameters (Table 1).

The mean oven-dry biomass was 384.08 g m^−2^ for the branch component and 219.62 g m^−2^ for the leaf component, and the difference was statistically significant (*p* = 0.002). Similarly, dry matter content was significantly higher in branches (52.75%) than in leaves (38.35%) (*p* < 0.001). The mean aboveground carbon stock was estimated as 192.04 g C m^−2^ in branches and 109.81 g C m^−2^ in leaves, with a statistically significant difference between the two components (*p* = 0.002). Likewise, the CO_2_-equivalent value was 704.79 g CO_2_e m^−2^ for branches and 403.00 g CO_2_e m^−2^ for leaves, and this difference was also statistically significant (*p* = 0.002). When the branch and leaf components were considered together, the mean total aboveground oven-dry biomass was 603.70 g m^−2^, corresponding to an estimated total aboveground carbon stock of 301.85 g C m^−2^ and a total CO_2_-equivalent value of 1107.79 g CO_2_e m^−2^. Pearson correlation analysis revealed statistically significant relationships among the biomass and dry matter parameters (Table 2).

A very strong positive correlation was found between total dry biomass (TDB) and oven-dry branch biomass (ODB) (*r* = 0.928, *p* < 0.001). Similarly, oven-dry leaf biomass (ODL) was significantly and positively correlated with total dry biomass (TDB) (*r* = 0.648, *p* = 0.007). A strong positive correlation was identified between branch dry matter content (BDM) and total dry matter content (TDM) (*r* = 0.772, *p* < 0.001), while leaf dry matter content (LDM) was also significantly and positively correlated with total dry matter content (TDM) (*r* = 0.690, *p* = 0.003). Pearson correlation analysis revealed a very strong positive correlation between the leaf-to-branch ratio (LBR) and leaf mass fraction (LMF) (*r* = 0.988, *p* < 0.001). In contrast, a perfect negative correlation was observed between branch mass fraction (BMF) and LMF (*r* = −1.000, *p* < 0.001). In addition, LMF showed a strong positive relationship with oven-dry leaf biomass (ODL) (*r* = 0.639, *p* = 0.008), while LBR was also significantly and positively correlated with ODL (*r* = 0.605, *p* = 0.013).

A strong positive correlation was found between mean branch diameter (MBD) and total dry biomass (TDB) (*r* = 0.803, *p* < 0.001). Mean branch diameter was also strongly and positively correlated with oven-dry branch biomass (ODB), branch carbon stock (BC), and branch CO_2_ equivalent (BCO_2_) (*r* = 0.772, *p* < 0.001). Furthermore, a strong positive relationship was identified between mean branch diameter and total carbon stock (TC) (*r* = 0.803, *p* < 0.001).

The dry matter content parameters (BDM, LDM, and TDM) did not show statistically significant correlations with most of the biomass, carbon-stock, and CO_2_-equivalent variables examined (*p* > 0.05).

**Figure 1:**
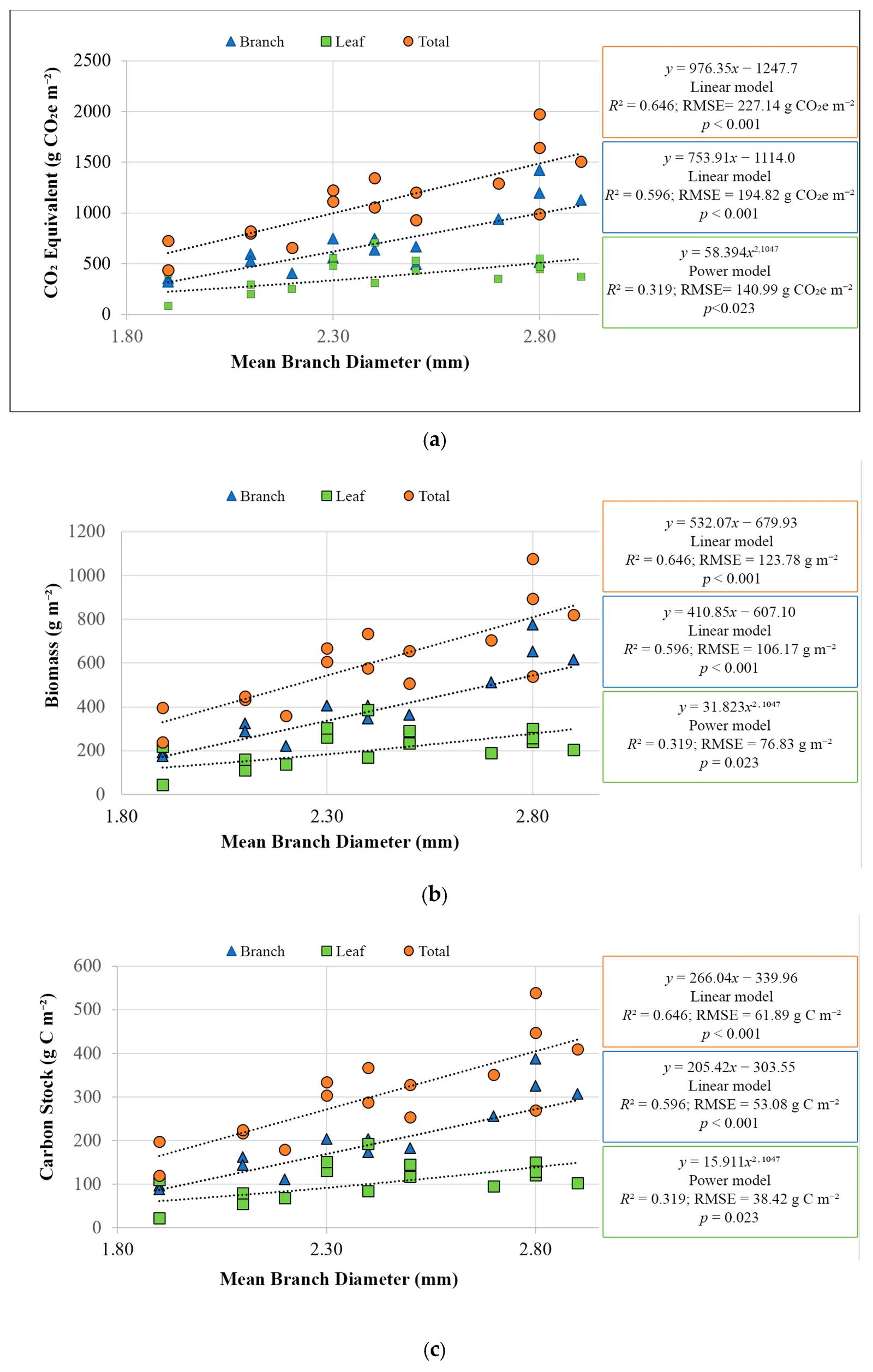
(**a**). Allometric relationships between mean branch diameter and branch, leaf, and total CO_2_ equivalent. (**b**). Allometric relationships between mean branch diameter and branch, leaf, and total biomass. (**c**). Allometric relationships between mean branch diameter and branch, leaf, and total carbon stock.

The linear models developed between mean branch diameter and branch biomass, branch carbon stock, and branch CO_2_ equivalent explained 59.6% of the variation (*R*^2^ = 0.596). The linear models developed for total biomass, total carbon stock, and total CO_2_ equivalent explained 64.6% of the variation (*R*^2^ = 0.646). In contrast, the models developed for leaf biomass, leaf carbon stock, and leaf CO_2_ equivalent had lower explanatory power (*R*^2^ = 0.319), and these variables were better described by a power function (Figure 1a–c). Residuals showed no significant departures from normality (*p* > 0.05); however, diagnostic tests indicated potential heteroscedasticity in the branch biomass and leaf biomass models and identified several potentially influential observations. Therefore, regression estimates were interpreted cautiously, particularly given the limited sample size (*n* = 16).

**Figure 2:**
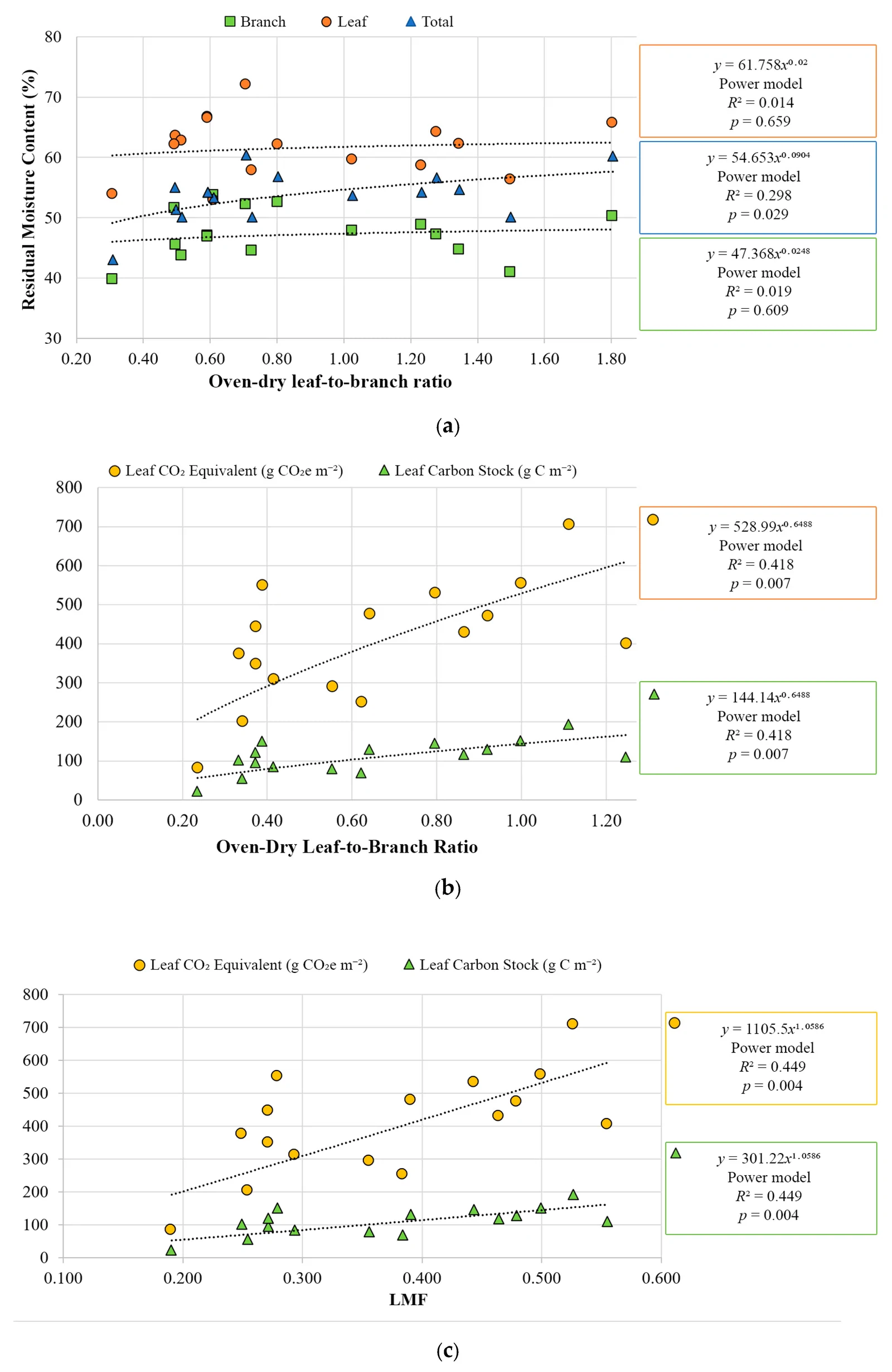

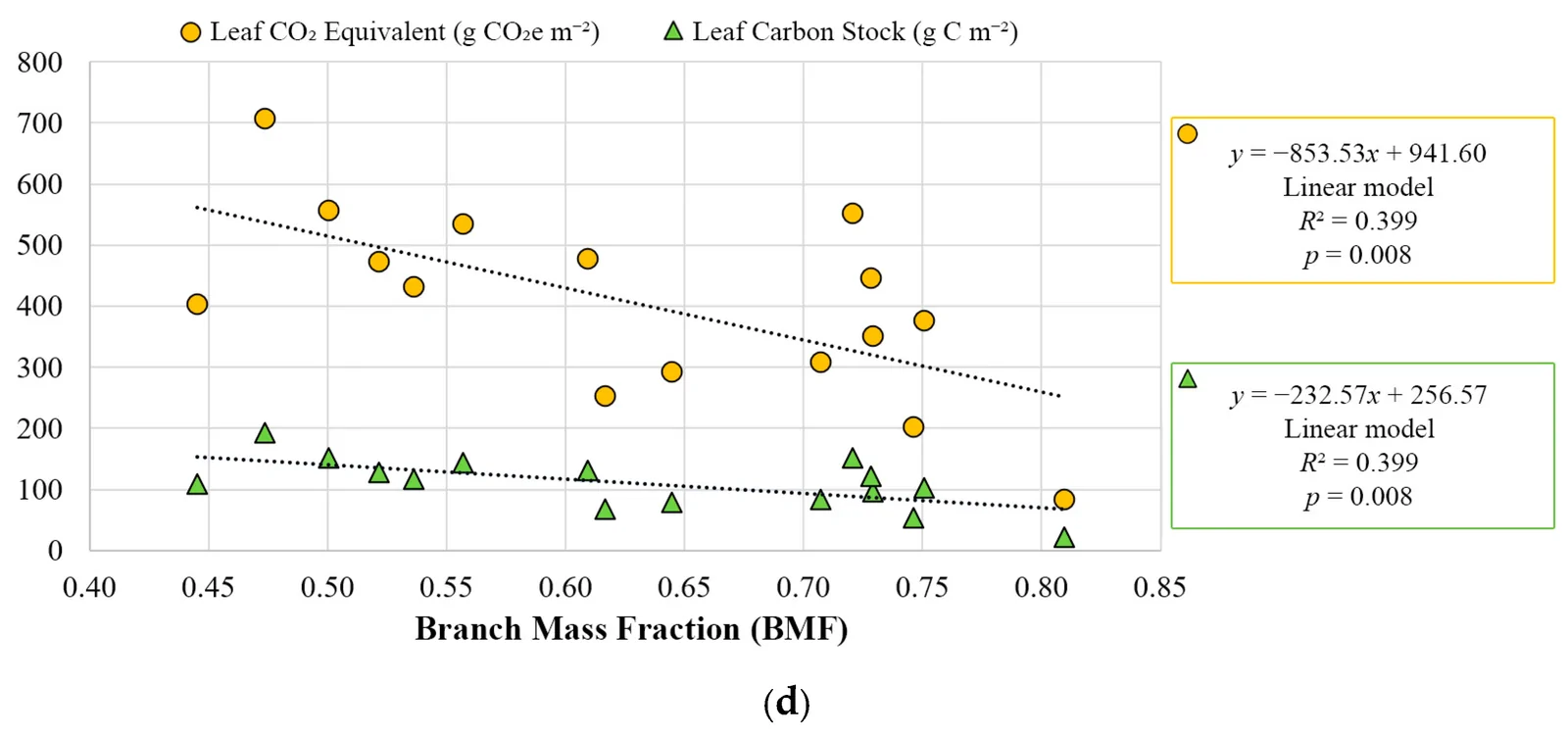
(**a**). Relationships between the oven-dry leaf-to-branch ratio and residual moisture content after air drying of branch, leaf, and total biomass. (**b**). Relationships between oven-dry leaf-to-branch ratio and leaf carbon stock and CO_2_ equivalent. (**c**). Relationships between leaf mass fraction (LMF) and leaf carbon stock and CO_2_ equivalent. (**d**). Relationships between branch mass fraction (BMF) and leaf carbon stock and CO_2_ equivalent.

For the relationships between residual moisture content after air drying and the oven-dry leaf-to-branch ratio, the power function provided the best-fitting model for all components (Figure 2a). A significant positive relationship was identified between total residual moisture content after air drying and the oven-dry leaf-to-branch ratio (*R*^2^ = 0.298, *p* = 0.029), whereas the models for branch and leaf residual moisture contents after air drying had very low explanatory power (*R*^2^ = 0.019 and 0.014, respectively).

Significant positive relationships were identified between the oven-dry leaf-to-branch ratio (LBR) and both leaf carbon stock and leaf CO_2_ equivalent (Figure 2b). For both variables, the correlation coefficient was *r* = 0.605 (*p* = 0.013). Regression analyses showed that these relationships were better described by a power function, with the resulting models having an explanatory power of *R*^2^ = 0.418.

Significant positive relationships were identified between leaf mass fraction (LMF) and both leaf carbon stock and leaf CO_2_ equivalent (Figure 2c). For both variables, the correlation coefficient was *r* = 0.639 (*p* = 0.008). Regression analyses showed that these relationships were better described by a power function, and the resulting models had an explanatory power of *R*^2^ = 0.449.

Significant negative relationships were identified between branch mass fraction (BMF) and both leaf carbon stock and leaf CO_2_ equivalent (Figure 2d). The correlation coefficients were identical for leaf carbon stock and leaf CO_2_ equivalent (*r* = −0.639, *p* = 0.008). Regression analyses showed that these relationships were better described by a linear model, with the resulting models having an explanatory power of *R*^2^ = 0.399.

Overall, mean branch diameter showed the strongest relationships with biomass and the corresponding derived carbon-stock and CO_2_-equivalent indicators among the variables examined in this study. The leaf-to-branch ratio (LBR), leaf mass fraction (LMF), and branch mass fraction (BMF), which represent biomass allocation, showed statistically significant relationships with leaf carbon stock and the corresponding CO_2_ equivalent. However, these relationships should be interpreted cautiously because LBR and LMF contain leaf biomass in their calculation, while leaf carbon stock and CO_2_ equivalent are directly derived from leaf biomass. Therefore, part of the observed associations is mathematically expected rather than representing independent ecological effects. In addition, LMF and BMF are complementary fractions that sum to unity and should not be considered independent variables. In contrast, no significant relationships were identified between residual moisture content after air drying and carbon-storage indicators.

## 3. Discussion

In this study, the biomass accumulation, aboveground carbon stock, and CO_2_-equivalent values of *Hedera colchica* growing on vegetated retaining walls were determined, and the distribution of carbon between branch and leaf components was evaluated.

The findings revealed substantial differences among the plant organs of *Hedera colchica* in terms of biomass accumulation and aboveground carbon storage. Branch components had higher biomass, carbon stock, and CO_2_-equivalent values than leaves and therefore contributed more to total carbon storage. Strong positive relationships were also identified between mean branch diameter and biomass, carbon stock, and CO_2_-equivalent values. The present findings indicate that carbon storage in retaining walls covered with *Hedera colchica* occurs predominantly in woody tissues. This allocation pattern may therefore reflect a biomass-investment strategy in which the perennial woody framework of *H. colchica* functions not only as structural support for vertical growth but also as a relatively persistent aboveground carbon pool.

In this study, branch biomass was significantly higher than leaf biomass. Similarly, the contributions of branch components to total carbon stock and CO_2_-equivalent values were greater than those of leaves. This pattern may be associated with the perennial and woody growth habit of *Hedera colchica*. In woody climbing plants, structural tissues provide mechanical support for vertical growth and may also function as relatively persistent carbon pools. By contrast, although leaves are the primary organs responsible for photosynthetic activity, their lower dry matter proportion and periodic replacement limit their contribution to long-term carbon storage. The higher dry matter content recorded in the branch components in the present study supports this interpretation. Previous studies have similarly reported that a substantial proportion of carbon in woody plants is stored in long-lived tissues such as stems and branches [23,41,42,43].

The aboveground dry biomass recorded for *Hedera colchica* in the present study (603.70 g m^−2^) can be compared with values reported for other *Hedera* systems. Blinkova et al. (2024) reported mean dry leaf and stem biomass values of 103.21 and 114.95 g m^−2^, respectively, for vegetative shoots of *H. helix*, corresponding to a combined leaf and stem dry biomass of 218.16 g m^−2^ [44]. Thus, the aboveground dry biomass recorded for *H. colchica* in the present study was substantially higher. However, direct species-level comparisons should be made cautiously because the studies differed in climatic conditions, habitat characteristics, plant developmental status, and sampling design. At the organ level, the greater contribution of woody tissues to carbon storage observed in the present study is consistent with Mirhosseini et al. (2024), who reported significant differences in carbon sequestration among the organs of *H. colchica*, with stems representing the largest carbon pool [45]. Together, these findings indicate that biomass accumulation and carbon allocation in *Hedera* may vary considerably among species, developmental stages, and environmental conditions.

The distribution of carbon stock among plant organs followed a pattern similar to that of biomass allocation, with carbon stock and CO_2_-equivalent values being significantly higher in branch components than in leaves. This pattern may be associated with differences in tissue characteristics and longevity: although leaves play a fundamental role in the photosynthetic uptake of atmospheric carbon, their contribution to long-term carbon storage is limited by their lower dry matter content and relatively short lifespan, whereas branches retain carbon within the system for longer periods because of their higher dry matter content and greater longevity [41,42]. These findings suggest that assessments of the carbon-storage potential of vertical greening systems should consider not only total biomass but also its allocation among plant organs. Because the aboveground carbon-stock estimates were based on a fixed carbon fraction of 0.50, a simple sensitivity assessment was also considered to illustrate the effect of uncertainty in this coefficient. Based on the observed mean oven-dry biomass values, use of a carbon fraction of 0.45 would result in estimated branch and leaf carbon stocks of 172.84 and 98.83 g C m^−2^, respectively, corresponding to a total aboveground carbon stock of 271.67 g C m^−2^. In contrast, use of a carbon fraction of 0.55 would result in branch and leaf carbon stocks of 211.24 and 120.79 g C m^−2^, respectively, corresponding to a total of 332.04 g C m^−2^. The corresponding total carbon stock obtained using the adopted fraction of 0.50 was 301.85 g C m^−2^. Thus, changing the assumed carbon fraction from 0.50 to 0.45 or 0.55 would decrease or increase the estimated carbon stock by approximately 10%, respectively, while leaving the relative biomass-based pattern between branch and leaf components unchanged. This sensitivity emphasizes that the absolute carbon-stock values should be interpreted as coefficient-based estimates until species- and organ-specific carbon fractions are determined directly. However, the relationships involving LBR, LMF, and BMF should not be interpreted as independent ecological predictors of carbon storage. Because these allocation indices are calculated directly from branch and leaf biomass, and carbon stock and CO_2_-equivalent values are themselves derived from biomass using fixed conversion factors, some of the observed correlations are inherently affected by mathematical coupling. Accordingly, these relationships are interpreted here primarily as descriptive indicators of biomass allocation rather than as evidence of independent causal effects on carbon storage.

The absence of significant relationships between residual moisture content and aboveground carbon-stock indicators may be explained partly by the fact that carbon stock was estimated from oven-dry biomass, thereby excluding the direct contribution of water remaining in the air-dried samples. It should be emphasized that the residual moisture variable used in the present study was calculated from air-dry and oven-dry masses and therefore does not represent field moisture content or plant water status at the time of harvesting. Consequently, the observed relationships should be interpreted only in the context of residual moisture remaining after the standardized air-drying procedure.

A strong positive relationship was observed between mean branch diameter and biomass, carbon stock, and CO_2_-equivalent values. As mean diameter increased, biomass and the associated carbon indicators also increased. This relationship may be explained by the close association of diameter growth with woody tissue development and biomass accumulation. The correlation and regression analyses indicate that mean branch diameter may serve as a simple, site-specific morphological indicator of aboveground biomass under the conditions examined in this study. However, because branch diameter was measured after harvesting in the present study, its potential application as a non-destructive field-based estimator should be validated in future studies using pre-harvest diameter measurements. However, further validation across different sites, plant ages, and architectural characteristics is required before broader application. Similarly, diameter measurements are widely used in forestry and carbon inventory studies to estimate biomass and carbon stocks [46,47,48].

The findings suggest that vegetated retaining walls may serve as complementary components of urban green infrastructure, particularly by contributing to aboveground carbon storage under suitable site conditions. Particularly in cities with sloping topography, retaining walls occupy extensive surface areas but are often treated solely as engineering structures and are not adequately evaluated in terms of the ecosystem services they may provide. The considerable biomass and carbon stock recorded for *Hedera colchica* in this study indicate that vegetated retaining walls may contribute to urban carbon storage.

Previous studies have also shown that vertical greening systems can provide a range of environmental benefits, including reduced surface temperatures, support for biodiversity, and improved visual landscape quality [27,29,35,36,37,38,39,49]. In particular, *Hedera colchica*, which is well adapted to the humid climatic conditions of the study region, may represent a suitable nature-based solution for increasing urban vegetation cover without requiring additional horizontal land area.

The environmental conditions of the study area may have influenced the observed biomass and carbon-stock values. *Hedera colchica* exhibits strong vegetative growth under the humid and temperate climatic conditions within its natural distribution range. The high relative humidity and regular rainfall characteristic of the Eastern Black Sea Region may have provided favorable conditions for plant growth and biomass accumulation. Accordingly, the substantial biomass and carbon stock observed in this study may partly reflect the favorable environmental conditions of the study site. Further studies across different climatic and site conditions are needed to determine the extent to which these findings can be generalized to other vertical green infrastructure systems.

Nevertheless, the findings should be interpreted within the scope and limitations of the study. The research was conducted on three adjacent retaining-wall blocks within a single study site and during a single sampling period. Because the sampling quadrats were distributed among wall blocks located within the same site, some degree of spatial autocorrelation among observations cannot be completely excluded. Accordingly, the biomass and aboveground carbon-stock values reported in this study should be interpreted as site- and age-specific estimates rather than as broadly applicable values for *H. colchica* across different vertical greening systems. Future studies involving different climbing species, plant age classes, retaining-wall types, climatic regions, and repeated sampling periods would contribute to a more comprehensive understanding of biomass accumulation and carbon-stock dynamics. Overall, the present findings indicate that retaining walls covered with *H. colchica* can contribute to aboveground carbon storage under the humid climatic and site conditions examined in this study. However, given the single-site design, single sampling period, and specific plant age represented in this study, the magnitude and consistency of this contribution should be validated across different climatic regions, retaining-wall types, plant age classes, and repeated sampling periods before broader generalizations are made.

## 4. Materials and Methods

### 4.1. Study Area

The study area is located within the Main Campus of Recep Tayyip Erdoğan University in Rize Province, northeastern Türkiye. It is situated at approximately 41°02′14.75″ N latitude and 40°29′31.17″ E longitude, on north-facing terrain ranging from 15 to 100 m above sea level. The study area lies within the Eastern Black Sea Region, where *Hedera colchica* occurs naturally, and is characterized by high humidity and abundant rainfall. According to long-term climatic records, the mean annual temperature is 14.5 °C, the mean annual precipitation is 2292 mm, and the mean annual number of rainy days is 171.6 [50]. Based on the Thornthwaite climate classification, the study area belongs to the A B′1 r a′ climate type and exhibits very humid, mesothermal, oceanic climatic conditions without a water deficit [51]. These climatic conditions provide a suitable environment for the growth of *Hedera colchica*, which requires high atmospheric humidity.

### 4.2. Sampling Design

The study site comprised three physically separated stone retaining-wall blocks vegetated with *Hedera colchica*. The first wall block was approximately 4 m long and 1.50 m high, the second was 7 m long and 1.70 m high, and the third was 5 m long and 1.50 m high, corresponding to a total wall surface area of approximately 25.4 m^2^. Approximately two-thirds of the total wall surface was covered by *H. colchica*, corresponding to an estimated vegetated surface area of approximately 16.9 m^2^. The three wall blocks were separated by 1.60 m wide stair openings. All walls were vertically oriented, north-facing, and constructed of stone with a naturally rough and irregular surface texture. They were characterized by similar solar exposure and drainage conditions, and no trees, buildings, or other major shading elements occurred immediately in front of the sampled wall surfaces.

*Hedera colchica* plants had been intentionally planted during the same period in the natural soil located along the upper edges of the retaining walls and were approximately 7 years old at the time of sampling. The plants grew downward from the upper soil surface and formed cascading vegetation cover over the stone wall faces. No pots, planting pockets, modular panels, geotextile layers, artificial growing substrates, or supplemental irrigation systems were used. Plant water supply depended exclusively on natural precipitation under the humid and high-rainfall climatic conditions of Rize.

Plant sampling was conducted on 11 November 2025. Because vegetation cover was not completely continuous across the wall surfaces, a systematic longitudinal sampling framework was applied within areas of homogeneous *H. colchica* cover. Each wall block was divided into consecutive 1 m longitudinal sections, and one 40 × 40 cm sampling quadrat was located within the representative homogeneous vegetation cover of each section. Accordingly, 4 quadrats were sampled from the first wall block, 7 from the second, and 5 from the third, resulting in a total of 16 quadrats. This design provided a nominal longitudinal sampling interval of approximately 1 m while distributing the samples across the full length of the three wall blocks. Each quadrat represented an area of 0.16 m^2^. All aboveground biomass occurring within each quadrat was harvested and collected for laboratory analysis.

### 4.3. Laboratory Analyses

After harvesting, branch diameter measurements were performed on fresh plant material before the drying procedures. For each quadrat, a total of 12 branches were measured, with three branches selected from each of the four sides of the 40 × 40 cm quadrat. Branch diameter was measured approximately 1 cm above the cut point using a digital caliper (1108-150, INSIZE Co., Ltd., Suzhou, China) with a precision of 0.01 mm. The mean branch diameter (MBD) for each quadrat was calculated as the arithmetic mean of the 12 individual diameter measurements.

After the plant samples were transported to the laboratory, they were separated into branch and leaf fractions. To determine their air-dry weights, the samples were dried at room temperature under laboratory conditions until a constant weight was reached, which took approximately 20 days. The samples were then oven-dried (WF-HT125, Weightlab Instruments, Istanbul, Türkiye) at 70 °C for 48 h until a constant weight was obtained, and their oven-dry weights were recorded [52,53].

### 4.4. Calculation of Biomass Parameters

The air-dry and oven-dry weights of branch, leaf, and total biomass were converted to unit-area values (g m^−2^) based on the sampling area of 0.16 m^2^. Using the calculated biomass values, residual moisture content after air drying (Equation (1)), leaf and branch dry matter contents (Equations (2) and (3)), leaf mass fraction (LMF; Equation (4)), and branch mass fraction (BMF; Equation (5)) were calculated as follows:

(1) $$\mathrm{Residual}\;\mathrm{moisture}\;\mathrm{content}\;\mathrm{after}\;\mathrm{air}\;\mathrm{drying}\;(\%)\;=\;\frac{\left(\mathrm{Air}-\mathrm{Dry}\;\mathrm{Sample}\;\mathrm{Weight}-\mathrm{Oven}-\mathrm{Dry}\;\mathrm{Sample}\;\mathrm{Weight}\right)}{\mathrm{Air}\;\mathrm{Dry}\;\mathrm{Sample}\;\mathrm{Weight}}\times 100$$

(2) $$\mathrm{Leaf}\;\mathrm{Dry}\;\mathrm{Matter}\;\mathrm{Content}\;(\%)=(\frac{\mathrm{Oven}-\mathrm{Dry}\;\mathrm{Leaf}\;\mathrm{Weight}}{\mathrm{Air}-\mathrm{Dry}\;\mathrm{Leaf}\;\mathrm{Weight}})\times 100$$

(3) $$\mathrm{Branch}\;\mathrm{Dry}\;\mathrm{Matter}\;\mathrm{Content}\;(\%)=(\frac{\mathrm{Oven}-\mathrm{Dry}\;\mathrm{Branch}\;\mathrm{Weight}}{\mathrm{Air}-\mathrm{Dry}\;\mathrm{Branch}\;\mathrm{Weight}})\times 100$$

(4) $$\mathrm{Leaf}\;\mathrm{Mass}\;\mathrm{Fraction}\;(\mathrm{LMF},\;\%)=(\frac{\mathrm{Oven}-\mathrm{Dry}\mathrm{Leaf}\;\mathrm{Weight}}{\mathrm{Total}\;\mathrm{Oven}-\mathrm{Dry}\;\mathrm{Weight}})\times 100$$

(5) $$\mathrm{Branch}\;\mathrm{Mass}\;\mathrm{Fraction}\;(\mathrm{BMF},\;\%)=(\frac{\mathrm{Oven}-\mathrm{Dry}\mathrm{Branch}\mathrm{Weight}}{\mathrm{Total}\;\mathrm{Oven}-\mathrm{Dry}\;\mathrm{Weight}})\times 100$$

Because fresh weight was not recorded immediately after harvesting, field moisture content could not be determined. Therefore, the moisture-related variable used in this study represents the residual moisture remaining after the air-drying period, calculated from the difference between the air-dry mass measured immediately before oven drying and the oven-dry mass obtained after drying at 70 °C. Dry matter content was calculated separately for branch, leaf, and total biomass and is subsequently reported as branch dry matter content (BDM), leaf dry matter content (LDM), and total dry matter content (TDM), respectively. The LMF and BMF values were calculated to evaluate biomass allocation between leaf and branch components, whereas residual moisture content was used to characterize the moisture remaining after air drying, and BDM, LDM, and TDM were used to describe dry matter proportions under the standardized laboratory drying procedure.

### 4.5. Calculation of Aboveground Carbon Stock and CO_2_ Equivalent

Because a species- and organ-specific carbon fraction for *Hedera colchica* was not available, the default carbon fraction of 0.50 adopted from IPCC (2006) was used as a standardized approximation for estimating biomass carbon content [54]. Aboveground carbon stock was calculated by multiplying oven-dry biomass by this carbon fraction, as shown in Equation (6). However, actual carbon concentrations may differ between branch and leaf components due to differences in their chemical composition. Therefore, the use of a single default carbon fraction may introduce uncertainty into organ-specific carbon-stock estimates, and the resulting values should be interpreted as estimates rather than direct measurements of tissue carbon concentration.

*C* = *B*dry × *F*c, (6)

where:

C = Aboveground carbon stock (g C m^−2^), Bdry = oven-dry biomass (g m^−2^), and Fc = carbon fraction coefficient (0.50).

The CO_2_ equivalent of the carbon stored in branch and leaf biomass was calculated using Equation (7). Carbon stock was multiplied by the stoichiometric conversion factor of 3.67 (44/12, rounded to two decimal places), which represents the ratio of the molar mass of CO_2_ to the atomic mass of carbon. In this conversion, 44 represents the molar mass of CO_2_ and 12 represents the molar mass of carbon:

CO_2_ equivalent = *C* × (44/12) ≈ *C* × 3.67 (7)

Because aboveground carbon stock and CO_2_ equivalent were derived directly from oven-dry biomass using fixed conversion factors, these variables were treated as derived indicators rather than independent response variables. Consequently, identical or proportional statistical relationships among biomass, carbon stock, and CO_2_ equivalent were interpreted as expressions of the same underlying biomass pattern.

### 4.6. Statistical Analyses

In this study, biomass, residual moisture content after air drying, dry matter content, carbon stock, and CO_2_-equivalent parameters of the branch and leaf components of *Hedera colchica* were evaluated using statistical methods. Differences between the branch and leaf components were analyzed using an independent-samples *t*-test. Before the analyses, the normality of the data was assessed based on skewness and kurtosis values, while homogeneity of variance was examined using Levene’s test. As the variables were normally distributed, the relationships between biomass characteristics and carbon-stock indicators were determined using Pearson correlation analysis. The relationships between mean branch diameter and biomass, carbon stock, and CO_2_-equivalent parameters were evaluated using linear and nonlinear regression models, including a power function. Model performance was evaluated using the coefficient of determination (*R*^2^) and root mean square error (RMSE). Regression diagnostics included assessment of residual normality using the Shapiro–Wilk test, homoscedasticity using the Breusch–Pagan test, and potentially influential observations using Cook’s distance. Statistical significance was accepted at *p* < 0.05, and all analyses were performed using IBM SPSS Statistics for Windows, Version 25.0 (IBM Corp., Armonk, NY, USA).

## 5. Conclusions and Recommendations

This study quantified aboveground biomass, aboveground carbon stock, and CO_2_-equivalent values at the plant-organ level in vegetated retaining walls covered with *Hedera colchica* and evaluated the relationships among these parameters. Branch components accounted for greater biomass, dry matter content, carbon stock, and CO_2_-equivalent values than leaves, highlighting the importance of perennial woody tissues as relatively persistent aboveground carbon pools. Mean branch diameter showed the strongest relationships with biomass and the corresponding derived carbon-stock and CO_2_-equivalent indicators, whereas the leaf-to-branch ratio (LBR), leaf mass fraction (LMF), and branch mass fraction (BMF) were particularly associated with leaf carbon stock. These findings indicate that aboveground carbon storage in *H. colchica* is closely associated with both total biomass and its allocation among plant organs.

By providing quantitative, organ-level estimates of aboveground biomass, carbon stock, and CO_2_ equivalent, this study contributes to the limited empirical information available for *H. colchica* in vertical greening systems. Under the humid climatic conditions examined, retaining walls covered with *H. colchica* showed measurable aboveground biomass and associated carbon stock, indicating that this species can contribute to carbon-storage ecosystem services under specific site conditions. These findings should not, however, be interpreted as evidence of a general multifunctional role of vegetated retaining walls, as other ecosystem services were not directly assessed in the present study.

Because the study was conducted at a single site, during a single sampling period, and on approximately 7-year-old plants, the reported biomass and carbon-stock values should be regarded as site- and age-specific estimates. Further research across different climatic regions, retaining-wall types, plant age classes, management conditions, and repeated sampling periods is needed to determine the robustness and broader applicability of these findings. Direct determination of species- and organ-specific carbon fractions through elemental analysis would further improve the accuracy of carbon-stock estimates, while comparative studies among *Hedera* species would help clarify species-specific differences in biomass allocation and carbon-storage potential.

## Figures and Tables

**Table 1:** Biomass, dry matter content, carbon stock, and CO_2_-equivalent values of the branch and leaf components of *Hedera colchica*.

Parameters	Plant Component	*n*	$\overset{¯}{x}$ ± SD	*t*-Value	*p*-Value
Oven-dry biomass (g m^−2^)	Branch	16	384.08 ± 172.55	3.426	*p* = 0.002
	Leaf	16	219.62 ± 84.23		
Dry matter content (%)	Branch	16	52.75 ± 4.11	8.857	*p* < 0.001
	Leaf	16	38.35 ± 5.04		
Aboveground carbon stock (g C m^−2^)	Branch	16	192.04 ± 86.28	3.426	*p* = 0.002
	Leaf	16	109.81 ± 42.11		
CO_2_ equivalent (g CO_2_e m^−2^)	Branch	16	704.79 ± 316.64	3.426	*p* = 0.002
	Leaf	16	403.00 ± 154.56		

Note: Values are presented as mean ($\overset{¯}{x}$) ± standard deviation (SD), *n* = 16.

**Table 2:** Pearson correlation matrix for biomass, dry matter content, biomass allocation, and mean branch diameter variables in *Hedera colchica*.

Variables	ODB	ODL	TDB	BDM	LDM	TDM	LBR	LMF	BMF	MBD
ODB	1	0.318	0.928 **	−0.184	−0.409	−0.125	−0.497	−0.472	0.472	0.772 **
ODL	0.318	1	0.648 **	−0.138	−0.065	−0.368	0.605 *	0.639 **	−0.639 **	0.467
TDB	0.928 **	0.648 **	1	−0.202	−0.354	−0.245	−0.162	−0.129	0.129	0.803 **
BDM	−0.184	−0.138	−0.202	1	0.306	0.772 **	−0.083	−0.142	0.142	−0.086
LDM	−0.409	−0.065	−0.354	0.306	1	0.690 **	0.136	0.144	−0.144	−0.390
TDM	−0.125	−0.368	−0.245	0.772 **	0.690 **	1	−0.384	−0.416	0.416	−0.216
LBR	−0.497	0.605 *	−0.162	−0.083	0.136	−0.384	1	0.988 **	−0.988 **	−0.204
LMF	−0.472	0.639 **	−0.129	−0.142	0.144	−0.416	0.988 **	1	−1.000 **	−0.150
BMF	0.472	−0.639 **	0.129	0.142	−0.144	0.416	−0.988 **	−1.000 **	1	0.150
MBD	0.772 **	0.467	0.803 **	−0.086	−0.390	−0.216	−0.204	−0.150	0.150	1

Abbreviations: ODB, oven-dry branch biomass; ODL, oven-dry leaf biomass; TDB, total dry biomass; BDM, branch dry matter content; LDM, leaf dry matter content; TDM, total dry matter content; LBR, leaf-to-branch ratio; LMF, leaf mass fraction; BMF, branch mass fraction; MBD, mean branch diameter. * Correlation is significant at the 0.05 level; ** correlation is significant at the 0.01 level.

## Data Availability

The original contributions presented in the study are included in the article. Further inquiries can be directed to the corresponding author.

## Abbreviations

The following abbreviations are used in this manuscript:

CO_2_	Carbon Dioxide
Fc	Carbon Fraction Coefficient
IPCC	Intergovernmental Panel on Climate Change
LBR	Leaf-to-Branch Ratio
LDM	Leaf Dry Matter Content
LMF	Leaf Mass Fraction
MBD	Mean Branch Diameter
ODB	Oven-Dry Branch Biomass
ODL	Oven-Dry Leaf Biomass
*R*^2^	Coefficient of Determination
RMSE	Root Mean Square Error
SD	Standard Deviation
SPSS	Statistical Package for the Social Sciences
TC	Total Carbon Stock
TDB	Total Dry Biomass
TDM	Total Dry Matter Content
